# Bisphosphonate Therapy and the Occurrence of the Hungry Bone Syndrome After Surgery for Primary Hyperparathyroidism

**DOI:** 10.1155/ije/8285521

**Published:** 2025-10-21

**Authors:** Rahil Etemadi, Asieh Mansouri, Rezvan Salehidoost, Elham Tavousi Tabatabaei

**Affiliations:** ^1^Endocrinology and Metabolism Research Center, Isfahan University of Medical Sciences, Isfahan, Iran; ^2^Department of Biostatistics and Epidemiology, Isfahan University of Medical Sciences, Isfahan, Iran; ^3^Department of Dermatology, Wayne State University School of Medicine, Detroit, Michigan, USA

**Keywords:** bisphosphonate, hungry bone syndrome, hyperparathyroidism, hypocalcemia, parathyroid adenoma, parathyroidectomy, primary hyperparathyroidism

## Abstract

**Objective:**

Hungry bone syndrome (HBS), marked by severe and persistent hypocalcemia, frequently occurs after parathyroidectomy for primary hyperparathyroidism (PHPT). Despite its prevalence, there is limited research on this complication. This study aimed to assess the impact of preoperative bisphosphonate treatment on the incidence of postoperative HBS in PHPT patients.

**Design:**

This retrospective study analyzed hospital records of patients with PHPT who underwent parathyroidectomy from January 2010 to January 2020.

**Patients:**

The study included 144 patients with PHPT who underwent curative parathyroidectomy at AL-Zahra University Hospital within the specified timeframe. Patients with secondary or tertiary hyperparathyroidism or unsuccessful surgeries were excluded.

**Measurements:**

Data on bisphosphonate use, clinical, and laboratory parameters were reviewed. Logistic regression analyzed the relationship between preoperative bisphosphonate treatment and the occurrence of postoperative HBS.

**Results:**

Of the total of 144 patients, 19 received preoperative bisphosphonate therapy. The incidence of HBS was significantly higher in the bisphosphonate group (57.9%) compared to the nontreated group (11.2%) (*p* < 0.001). However, logistic regression analysis revealed no significant reduction in HBS occurrence due to bisphosphonate treatment, either in the overall cohort or in patients with moderate to severe hypercalcemia (calcium level ≥ 12 mg/dL) (odds ratio: 3.4, 95% CI: 0.5–22.7, *p*=0.191; odds ratio: 15.0, 95% CI: 0.6–383.9, *p*=0.102, respectively).

**Conclusion:**

Preoperative bisphosphonate therapy does not prevent the development of HBS following parathyroidectomy for PHPT. Continuous postoperative calcium monitoring is essential for effective management and mitigation of HBS.

## 1. Introduction

Primary hyperparathyroidism (PHPT) represents one of the most frequent endocrine conditions, defined by excessive parathyroid hormone (PTH) secretion, resulting in hypercalcemia and hypophosphatemia [1]. Approximately 80–85% of patients with PHPT have a single adenoma, while the remaining cases are due to double adenomas, multiple gland hyperplasia, or, rarely, carcinoma [2]. Definitive therapy for PHPT involves surgical excision of the diseased parathyroid gland(s), thereby correcting the hormonal imbalance [2, 3]. Despite the effectiveness of surgery, postoperative hypocalcemia is a frequent complication, often due to functional hypoparathyroidism or hungry bone syndrome (HBS) [2, 4].

HBS occurs as a result of the high turnover state in bones associated with PHPT. Following parathyroidectomy, the abrupt removal of PTH stimulus leads to a reduction in bone resorption and an increase in bone formation, causing excess calcium and phosphorus to shift into the bones. HBS is characterized by severe, prolonged, and occasionally life-threatening hypocalcemia [2]. Severe hypocalcemia is a medical emergency that can result in seizures, cardiac arrhythmias, and laryngospasm, which are potentially life-threatening [5, 6]. HBS also leads to longer hospital stays and increased healthcare costs [7, 8].

The potential role of preoperative bisphosphonate therapy in reducing the risk of HBS has been explored in some studies, but results are inconsistent and often derived from small sample sizes [9–13].

Because HBS substantially influences both clinical outcomes and healthcare resources, we investigated the effectiveness of preoperative bisphosphonate treatment in preventing postoperative HBS in patients undergoing parathyroidectomy. By analyzing hospital records from patients with PHPT, this research seeks to address gaps in current knowledge and provide insights into improving clinical management.

## 2. Materials and Methods

This retrospective database study was conducted at AL-Zahra University Hospital. Hospital records of patients undergoing parathyroidectomy from January 2010 to January 2020 were extracted through continuous enrollment. Information was collected based on patients' files and hospital charts. The inclusion criterion was admission for parathyroid surgery. Patients with MEN, parathyroid carcinoma, secondary or tertiary hyperparathyroidism, or unsuccessful surgery were excluded. Individuals who had received preoperative calcitonin or cinacalcet were also omitted, since the study was designed to evaluate the effect of bisphosphonate therapy on HBS, and these drugs could act as confounders. The final cohort therefore included only patients with PHPT who underwent curative parathyroidectomy.

From the medical charts we obtained, were documented, demographic details (age and sex), past medical history of diabetes and hypertension, blood pressure at the time of admission, preoperative treatment with bisphosphonates (pamidronate or zoledronic acid), and preoperative and postoperative laboratory parameters, including total serum calcium, inorganic phosphorus, albumin, magnesium, kidney function tests (blood urea nitrogen and creatinine), PTH, potassium, and sodium. We defined sex as a biological attribute based on patients' self-reported information at the time of hospital admission. Gender-specific data beyond biological sex were not specifically assessed in this retrospective study. The distribution of male and female patients was determined based on patient records collected throughout the study.

The diagnosis of PHPT was made by identifying elevated serum calcium levels in the context of nonsuppressed PTH levels [14]. Corrected calcium levels were calculated using the following formula based on measured serum albumin and calcium levels: Corrected calcium (mg/dL) = (0.8 × [4 − patient's albumin [g/dL]]) + measured serum calcium level (mg/dL) [15].

To monitor metabolic changes after surgery, serum calcium, phosphorus, and albumin were checked each day throughout the hospitalization. Hypocalcemia was defined as a postoperative calcium level of ≤ 8.5 mg/dL. The size and weight of the parathyroid adenoma were determined by pathological examination. Tumor volume was derived from the product of its three axes, corrected with the conventional constant used for ellipsoid structures. Hospital stay duration was defined as the interval between admission and discharge dates.

Postoperative care for hypocalcemic patients included treatment with oral or intravenous calcium to maintain serum calcium levels at 8.5 mg/dL or higher. Treatment for other postoperative electrolyte disorders, such as hypomagnesemia, was also provided when necessary. Information about the type of postoperative treatment was categorized into oral and intravenous treatments for both calcium and magnesium.

We classified patients as having HBS if the two conditions were met: (1) hypocalcemia, defined as serum calcium ≤ 8.5 mg/dL on or after the fourth postoperative day, or the need for calcium supplementation to avoid such a decline at that time point; and (2) concurrent hypophosphatemia, with serum phosphate < 3.0 mg/dL measured on the same day [4, 7].

The study was approved by the Medical Ethics Committee of Isfahan University of Medical Sciences (Isfahan research project number: IR.MUI.REC.1396.3.974). All participants provided written informed consent before enrollment.

### 2.1. Statistical Analysis

Statistical analyses were conducted using SPSS Version 25 (IBM Corp., Armonk, NY, USA) and STATA Version 12 (StataCorp LLC, College Station, TX, USA). Continuous data are summarized as mean (SD) when normally distributed and as median (IQR) when skewed. Categorical variables are expressed as counts and percentages. Group comparisons for normal and non-normal continuous variables between positive and negative HBS groups were performed using independent *t*-tests and Mann–Whitney *U* tests, respectively. Chi-square tests were used to compare categorical variables between these groups. Fisher's exact test was employed for categorical variables with small cell sizes. Logistic regression models were employed to assess the impact of bisphosphonate therapy on HBS occurrence. A sensitivity analysis was conducted by excluding patients with preoperative serum calcium levels less than 12 mg/dL, given the routine use of bisphosphonates in managing severe hypercalcemia.

## 3. Results

Out of the 144 patients included in our study, 106 (73.6%) were female and 38 (26.4%) were male. The mean (SD) age of all patients was 52.6 (12.6) years. HBS developed in 25 patients (17.36%), with no significant difference observed between genders (*p*=0.425). Nineteen patients received preoperative bisphosphonate treatment, comprising 5 with zoledronic acid and 14 with pamidronate. The number (%) of patients with HBS was 11/19 (57.9%) and 14/125 (11.2%) in the group treated preoperatively with bisphosphonate and without treatment with bisphosphonate in all patients, respectively (*p* ≤ 0.001). Among patients with serum calcium levels ≥ 12 mg/dL (*n* = 39), the incidence of HBS was 11/19 (57.9%) and 5/20 (25.0%) in the bisphosphonate-treated and untreated groups, respectively. The proportion of HBS was 9/105 (8.6%) in patients with serum calcium levels < 12 mg/dL. Patients with mild hypercalcemia did not receive bisphosphonate therapy.

Overall, 58 patients (40.3%) showed suppressed PTH after parathyroidectomy, defined as a postoperative PTH level < 15 pg/mL on the first day after surgery. Suppressed PTH was observed in 10 patients (52.6%) who had received preoperative bisphosphonates and in 48 patients (38.4%) who had not (Fisher's exact test, *p*=0.763).

Baseline clinical and biochemical characteristics are summarized in Table 1 according to bisphosphonate treatment status. The group receiving bisphosphonate had significantly higher baseline serum PTH levels and corrected calcium values but lower baseline serum magnesium and phosphorus levels.

Table 2 presents postoperative data among patients with PHPT who underwent surgery, stratified by preoperative bisphosphonate treatment. The bisphosphonate-treated group had significantly longer hospital stays and lower serum phosphorus levels immediately and on the first day after surgery. These patients also required significantly more oral and intravenous calcium and magnesium supplementation postsurgery. Serum calcium and magnesium levels immediately and on the first day after surgery did not differ significantly between the two groups.

We performed unadjusted and adjusted logistic regression to evaluate the effect of bisphosphonate pretreatment on HBS occurrence among PHPT patients undergoing parathyroidectomy (Table 3). The unadjusted model showed that bisphosphonate pretreatment significantly increased HBS occurrence. However, this effect was not confirmed after adjusting for known HBS risk factors from previous studies, including age, sex, baseline corrected serum calcium, inorganic phosphorus, magnesium, blood urea nitrogen, PTH levels, and parathyroid adenoma volume. This held true for all patients and for those with moderate to severe hypercalcemia (baseline calcium ≥ 12 mg/dL).

## 4. Discussion

HBS may occur in patients undergoing parathyroidectomy for PHPT, manifesting as persistent severe hypocalcemia. The incidence of HBS varies across studies. Western studies report up to 14% incidence following successful surgery for PHPT [7, 16], while studies from India show a much higher proportion [17]. In our study, HBS developed in 25 (17.36%) patients. The lower incidence in Western countries may be due to routine screening programs detecting mild disease in asymptomatic phases. In contrast, studies from India often include symptomatic patients with renal or bone diseases, potentially increasing the incidence of HBS [17]. Patients with preoperatively high serum calcium, PTH, alkaline phosphatase, and skeletal manifestations such as osteitis fibrosa cystica, brown tumors, and severe osteoporosis are at higher risk of developing HBS [7].

In PHPT, hypercalcemia results from increased bone turnover with excessive osteoclastic activity and increased renal tubular calcium reabsorption. After successful surgery, the sudden removal of PTH effect leads to enhanced osteoblastic activity, resulting in rapid bone calcium uptake and decreased serum calcium levels [2].

Bisphosphonate therapy is routinely used to correct severe hypercalcemia in the immediate preoperative phase. In our study, 19 patients received preoperative bisphosphonate treatment. The proportion of patients with HBS was as follows: 11/19 in patients with serum calcium levels of 12 mg/dL or more who received preoperative bisphosphonate treatment, 5/20 in patients with serum calcium levels of 12 mg/dL or more without bisphosphonate treatment, and 9/105 in patients with serum calcium levels less than 12 mg/dL. Our findings suggest that pretreatment with bisphosphonate did not decrease HBS occurrence.

Literature on bisphosphonate therapy for preventing HBS after surgery is limited and conflicting. Current data are largely based on retrospective observations of small patient populations or case reports. The effect of preoperative bisphosphonate therapy on HBS remains controversial. For instance, one case report indicated that HBS did not develop in a patient with severe PHPT treated with alendronate for six years and preoperative pamidronate (180 mg) intravenously [9]. A retrospective study found that HBS did not occur in six patients who received preoperative bisphosphonates (either oral clodronate or i.v. pamidronate) and concluded that bisphosphonates could prevent HBS postparathyroidectomy [10]. Another retrospective study reported a low frequency of postoperative HBS (4%) in 46 patients preoperatively treated with i.v. zoledronate but noted that bisphosphonate use did not completely prevent HBS [18]. Conversely, a case report described a young woman with PHPT who developed severe HBS postparathyroidectomy despite pretreatment with intravenous pamidronate [19]. Another report described a patient treated with zoledronic acid who developed severe postoperative hypocalcemia resistant to standard treatment, suggesting that bisphosphonates could exacerbate HBS in the preoperative phase [13].

Bisphosphonates are effective in controlling hypercalcemia due to PHPT, allowing time for localization studies. These agents, structurally similar to pyrophosphates, inhibit osteoclastic bone resorption and decrease bone remodeling. In hyperparathyroid bone disease, bisphosphonates inhibit bone resorption while coupled bone formation continues. Therefore, some studies suggest that short-term preoperative bisphosphonate treatment may not effectively control postoperative hypocalcemia. It has been hypothesized that bisphosphonates may only be beneficial when used long-term before parathyroidectomy and when alkaline phosphatase levels are normalized before surgery, although this theory has not been tested in randomized controlled trials.

In the present study, bisphosphonate use immediately before surgery did not prevent HBS in an adjusted model. This may be due to the inhibition of bone resorption without allowing sufficient time for a decrease in bone formation. In our study, bisphosphonates were administered to patients with higher calcium concentrations. We conducted a sensitivity analysis excluding patients with preoperative serum calcium levels less than 12 mg/dL, which showed that bisphosphonate therapy did not prevent HBS in patients with moderate to severe hypercalcemia.

This study has both strengths and limitations. It is among the first to assess the effect of bisphosphonate therapy on HBS in PHPT and represents one of the largest single-center cohorts reported to date. Nonetheless, its retrospective design and reliance on chart reviews limited the availability of certain data. Measurements such as bone-specific or total alkaline phosphatase, vitamin D status, severe osteoporosis, and fracture history were not consistently documented, and bisphosphonate use was not randomized. Although all patients with PHPT who underwent curative parathyroidectomy between January 2010 and January 2020 were included, the overall sample size remained modest due to the rarity of the disease. While this reduced the power to detect small differences, the findings still provide important insights and underscore the need for larger multicenter studies to clarify the relationship between bisphosphonate treatment and HBS.

In conclusion, our study demonstrated that preoperative bisphosphonate administration in patients with PHPT does not prevent HBS after parathyroidectomy. Therefore, postoperative calcium monitoring remains essential.

## Figures and Tables

**Table 1:** The preoperative baseline clinical and biochemical characteristics among patients with PHPT.

	Bisphosphonate pretreatment (*n* = 19)	No bisphosphonate pretreatment (*n* = 125)	*p* value
	Number, median, or mean (%, IQR or SD)	Number, median, or mean (%, IQR or SD)	
Age (years)	54.6 (14.1)	52.3 (12.4)	0.455^∗^
Gender (female)	15 (78.9)	91 (72.8)	0.571^+^
Diabetes (yes)	6 (31.6)	25 (20.0)	0.367^++^
Hypertension (yes)	6 (31.6)	47 (37.6)	0.612^+^
Systolic BP (mmHg)	125.4 (15.1)	124.6 (17.0)	0.845^∗^
Diastolic BP (mmHg)	76.8 (10.8)	77.0 (11.2)	0.935^∗^
Sodium (mg/dL)	141.0 (5.2)	140.8 (3.8)	0.846^∗^
Potassium (mg/dL)	4.1 (0.7)	4.3 (0.5)	0.241^∗^
BUN (mg/dL)	15 (10, 30)	15 (11, 20)	0.538^∗∗^
Creatinine (mg/dL)	1.2 (1.0, 1.6)	1.0 (0.9, 1.3)	0.074^∗∗^
Magnesium (mg/dL)	1.7 (1.6, 1.9)	1.9 (1.8, 2.1)	0.002^∗∗^
Calcium (mg/dL)	13.5 (12.4, 14.7)	11.0 (10.7, 11.7)	< 0.001^∗∗^
Albumin (g/dL)	4.0 (0.6)	4.2 (0.5)	0.339^∗^
Phosphorus (mg/dL)	2.3 (2.1, 2.6)	2.8 (2.5, 3.1)	0.002^∗∗^
PTH (pg/mL)	624 (314, 1312)	187 (128, 539)	< 0.001^∗∗^

Abbreviations: BP = blood pressure; BUN = blood urea nitrogen; IQR = interquartile range; SD = standard deviation.

^∗^Obtained from the independent sample *t*-test, ^∗∗^Obtained from the Mann–Whitney *U* test. ^+^Obtained from the chi-square test. ^++^Obtained from Fisher's exact test.

**Table 2:** Postoperative data among patients with PHPT who underwent parathyroidectomy.

	Bisphosphonate pretreatment (*n* = 19)	No bisphosphonate pretreatment (*n* = 125)	*p* value
	Number, median, or mean (%, IQR or SD)	Number, median, or mean (%, IQR or SD)	
Occurrence of HBS	11 (57.5)	14 (11.2)	< 0.001^+^
Length of stay (day)	12 (7, 19)	3 (2, 6)	< 0.001^∗^
Adenoma volume (mm^3^)	3.1 (1.3, 6.2)	1.3 (0.57, 3.58)	0.065^∗^
Adenoma weight (g)	6.5 (5.1, 9.5)	4.0 (2.5, 8.0)	0.094^∗^
PTH (pg/mL)	12.7 (10.6, 13.6)	13 (6.8, 17.7)	0.828^∗^
Ph on OP day (mg/dL)	2.2 (2.0, 2.5)	2.7 (2.4, 3.1)	< 0.001^∗^
Ph on 1^st^ post-OP (mg/dL)	2.1 (2.0, 2.2)	2.8 (2.2, 3.3)	< 0.001^∗^
Ca on OP day (mg/dL)	8.9 (8.0, 10.2)	9.3 (8.6, 9.9)	0.324^∗^
Ca on 1^st^ post-OP (mg/dL)	8.8 (8.2, 9.7)	8.9 (8.4, 9.2)	0.887^∗^
Magnesium (mg/dL)	1.7 (1.5, 1.8)	1.7 (1.6, 1.9)	0.448^∗^
PTW oral Ca (yes)	16 (84.2)	57 (45.6)	0.002^+^
PTW IV Ca (yes)	12 (63.1)	31 (24.8)	0.001^+^
PTW oral Mg (yes)	17 (89.5)	21 (16.8)	< 0.001^+^
PTW IV Mg (yes)	11 (57.9)	6 (4.8)	< 0.001^++^

Abbreviations: Ca = calcium; IQR = interquartile range; OP = operation; Ph = phosphorus; PTH = parathyroid hormone; PTW = postoperative treatment with; SD = standard deviation.

^∗^Obtained from the Mann–Whitney *U* test. ^+^Obtained from the chi-square test. ^++^Obtained from Fisher's exact test.

**Table 3:** Unadjusted and adjusted odds ratios for the occurrence of HBS according to preoperative treatment with bisphosphonate.

Variable	Odds ratio (95% CI)	*p* value
All patients		
Bisphosphonate (unadjusted)	10.9 (3.7–31.7)	< 0.001
Bisphosphonate (adjusted)^∗^	3.4 (0.5–22.7)	0.191
Patients with preoperative serum calcium ≥ 12 mg/dL		
Bisphosphonate (unadjusted)	7.1 (2.1–24.4)	0.002
Bisphosphonate (adjusted)^∗^	15.0 (0.6–383.9)	0.102

^∗^Models were adjusted for age, sex, baseline corrected serum calcium, baseline inorganic phosphorus, baseline magnesium, baseline blood urea nitrogen, baseline parathyroid hormone, and volume of parathyroid adenoma.

## Data Availability

The data supporting this study's findings will be made available by the corresponding author upon request.
